# Towards a Comprehensive Understanding of Body Image: Integrating Positive Body Image, Embodiment and Self-Compassion

**DOI:** 10.5334/pb.1057

**Published:** 2021-07-27

**Authors:** Diana Burychka, Marta Miragall, Rosa M. Baños

**Affiliations:** 1 Department of Personality, Evaluation and Psychological Treatment, University of Valencia, Spain; 2 Polibienestar Institute, University of Valencia, Valencia, Spain; 3 CIBER Fisiopatología Obesidad y Nutrición (CIBEROBN), Instituto Carlos III, Spain

**Keywords:** body image, positive embodiment, body shame, self-compassion

## Abstract

Body image (BI) disturbance is a relevant factor in the etiology and treatment of eating disorders (ED). Although progress has been made in recent decades in understanding BI and its relationship with ED, the efficacy of BI disturbance prevention and intervention programs is still limited. In order to reach deeper understanding of BI disturbance and clarify the interactions between some protective and risk factors related to this construct, we carried out a literature review on some specific BI-related factors that so far have been analyzed independently. We specifically examined positive and negative BI; embodiment and its role in the development of positive and negative BI; and self-compassion as a protective factor that promotes positive embodiment (vs. disembodiment) and protection against body shame. We conclude that integrating the available evidence on these factors into BI models may be used to enhance our understanding of BI and improve the efficacy of prevention and intervention programs to help fight negative BI (by reducing body shame and disembodiment) and promote positive BI (by increasing self-compassion and positive embodiment).

Body Image (BI) is a multidimensional concept that involves people’s positive and negative perceptions, thoughts, behaviors, and attitudes about their body and appearance (Gardner, 1996; Garner & Garfinkel, 1982, Grogan, 2016). The term was coined by Paul Schilder (1935), who defined BI as the mental representation of one’s body that everyone develops. The BI development process is dynamic, and it is influenced not only by the physical (e.g., body size or shape) or psychological (e.g., perfectionism, low self-esteem) characteristics of the individual, but also by the socio-cultural context (e.g., cultural ideal of beauty, media pressure to achieve an ideal of beauty) (Cash, 2002; Wertheim & Paxton, 2011). Moreover, BI is linked to the multifaceted psychological experience of embodying one’s body (Cash, 2004). Hence, BI is not only related to the way people perceive their body, but it also influences the way they interact with the world through that body (Piran & Teall, 2012).

BI disturbance (BID) has been identified as a key factor in the development and maintenance of eating disorders (ED) in general (Glashouwer et al., 2019; Mora-Giral et al., 2004; Stice & Shaw, 2002) and anorexia nervosa (Dakanalis et al., 2016) and bulimia nervosa (Degortes et al., 2018; Sattler et al., 2019), in particular. Furthermore, BID is also a crucial factor in the relapse and poor prognosis of these disorders (Bachner-Melman et al., 2006; Carter et al., 2004; Glashouwer et al., 2019), as well as their increasing prevalence (Mitchison et al., 2020), especially in the adolescent and young adult population (Treasure et al., 2010). Furthermore, even in the absence of an ED, BID is a risk factor that impacts the individual’s quality of life (Hosseini & Padhy, 2019).

Despite the large amount of research being conducted in the field, the efficacy of BI-focused interventions in ED remains limited (Alleva et al., 2015; Ziser et al., 2018). Particularly, interventions targeting BI only, lead to small improvement, highlighting the need for enhancing current therapeutic strategies (Alleva et al., 2015; Linardon et al., 2017, 2018; Linardon & Wade, 2018). Additionally, there is evidence that BID persists in patients with ED once the intervention is finished (Engel & Keizer, 2017; Eshkevari et al., 2014). Thus, it is necessary to consider other relevant BI-related protective and risk factors that may help improve existing assessment and intervention ED programs. For instance, there is evidence on the relationship between lower BID and higher level of positive embodiment (Cook-Cottone, 2015; Homan & Tylka, 2014) and higher levels of self-compassion (Braun et al., 2016). However, although over the last years these protective factors have gained prominence in the positive BI field (Braun et al., 2016), they have been explored independently (i.e., have not been integrated in explicative models of BID). Integrating these factors in more comprehensive explicative models may increase our understanding on the origin and maintenance of BID in patients with ED.

The aim of this paper was to carry out a narrative review of the existing literature on key protective and risk factors that are being related to higher positive BI and lower negative BI (i.e., sense of embodiment, self-compassion, and body shame). Specifically, this study will review: (1) positive and negative BI, (2) embodiment and its role in the development of positive and negative BI, and (3) self-compassion as a protective factor that promotes positive embodiment (vs disembodiment) and protects against body shame. Analysis of these factors may provide further insights into the complex construct of BI and help us to better understand their role in ED.

In this narrative review, we first analyze the traditional perspective, which is focused on negative BI. However, we also highlight the importance of positive BI (e.g., body acceptance), as well as its associated protective and risk factors. Thus, we first consider *positive embodiment* (vs. *disembodiment*) -a positive connection with one’s body- as a protective factor of positive and negative BI (Cook-Cottone, 2018). Embodiment, although considered for decades as relevant in this field (Cash, 2004), has been long overlooked and requires reconsideration to reach a more comprehensive understanding of BI. Second, we examine *body shame* ‒a self-conscious emotion that can disturb the connection to one’s body‒ (Piran & Neumark-Sztainer, 2020), a specific risk factor of negative BI in patients with ED and non-clinical ED samples (e.g., Ferreira et al., 2013; Duarte et al., 2015). Finally, we explore the role of *self-compassion* ‒the experience of understanding one’s own pain in a non-judgmental way and seeing suffering as a part of a shared human experience‒ (Neff, 2003), given its role in cultivating connection to one’s own body (positive embodiment) and positive BI (Braun et al., 2016). Self-compassion has emerged as a protective factor against body shame and disembodiment, and is one of the most effective intervention techniques in this field to reduce BID (Braun et al., 2016).

This review proposes that the integration of dimensions from positive and negative BI will result in a more comprehensive approach to BI. Therefore, the incorporation of factors associated to positive BI (i.e., positive embodiment, self-compassion), together with the extensively studied factors associated to negative BI (e.g., disembodiment, body shame), may improve not only the theoretical understanding of BI, but also lead to a development of specific therapeutic strategies to improve the intervention of BID and ensure long-lasting outcomes.

## The classic view of BI: negative BI and its dimensions

The BI construct seems to be composed of two dimensions: negative BI and positive BI. To date, research has focused primarily on the study of the negative dimension (Smolak & Cash, 2011; Tylka, 2011), characterized by BID. As noted above, BID is a key element in the expression of ED and one of the more common characteristics in anorexia nervosa and bulimia nervosa (Cash & Deagle, 1997; Cornelissen et al., 2013, 2015), as well as a key component in its development, maintenance, and relapse (Stice & Shaw, 2002; Treasure et al., 2020). Moreover, BID can also be found in the non-clinical population (McCabe et al., 2006; Stice & Whitenton, 2002), making its study and understanding even more relevant.

Regarding negative BI, the research has focused on the extensive examination of two independent subdimensions that can be disturbed (Garner & Garfinkel, 1982): (a) the perceptual dimension (which refers to the estimation of one’s body size and weight); and (b) the affective-attitudinal-cognitive dimension (which involves feelings, attitudes, and thoughts about one’s body size and weight) (Bulik et al., 2006). Perceptual disturbance is manifested as an underestimation or overestimation of body size or weight, whereas disturbance in the affective dimension is characterized mainly by body dissatisfaction and/or overvaluation of body size and weight (Cornelissen et al., 2013; Dakanalis et al., 2016). Therefore, disturbance can be found in one or both BI dimensions.

Most studies have prioritized the exploration of perceptual dimension disturbance. Currently, there is enough evidence to state there is a trend in patients with anorexia nervosa to be impaired in this dimension, characterized by greater overestimation of their perceived body size in comparison to control groups with no history of ED (Brown et al., 2021; Hagman et al., 2015; Gardner & Brown, 2014; Mölbert et al., 2017). This overestimation is likely to persist over time despite demanding diets and significant weight loss, which usually occurs in these patients (Riva et al., 2015). In the past few decades, the underlying mechanisms of this disturbance have been investigated in order to develop effective interventions to readjust body size estimation (Cornelissen et al., 2013).

Regarding the affective dimension of BI, several authors have emphasized its relevance, as well as its relationship with the perceptual dimension (Mölbert et al., 2018; Preston & Ehrsson, 2014, 2016). Overall, the evidence suggests that people with ED experience higher body dissatisfaction, greater concerns about body weight and/or size, an increased drive for thinness, and a lower desired weight, compared to people with no history of ED (Cash & Deagle, 1997; Moscone et al., 2017). In addition, studies have found that high levels of body dissatisfaction are associated with greater inaccuracy in one’s body size perception (Keizer et al., 2011), and that an increased drive for thinness is associated with greater overestimation of one’s body size (Hagman et al., 2015). Similarly, Gardner and Bokenkamp (1996) concluded that body dissatisfaction could be a causal factor in overestimating body size. Thus, there is a large body of research on the psychopathological symptoms associated with BI (e.g., Smolak, 2012; Thompson et al., 1999). In short, latest studies (Hagman et al., 2015; Mölbert et al., 2018) point out the importance of studying in depth the affective dimension of BI (e.g., body shame) to understand perceptual BID (e.g., body overestimation).

Additionally, although most of these studies have focused on the negative BI, new explanatory models of ED have recently been developed. The need to study the “positive” side of BI has emerged, leading to a better understanding of both the risk factors and the possible protective factors in the development of ED (Tylka, 2012).

## Positive BI: A necessary dimension for the comprehensive understanding of BI

Positive BI was initially defined as an opposite concept to negative BI (Smolak, 2012; Tylka, 2011, 2012), so that a reduction in BID was associated with an increase in positive BI characteristics (Tylka & Wood-Barcalow, 2015). Based on this approach, BI was originally considered a continuum with negative and positive BI situated at opposite ends (Webb et al., 2015). However, a growing body of evidence indicates that negative and positive BI are not opposite ends of the same continuum, but rather two different constructs that are negatively correlated (Avalos et al., 2005; Tylka, 2011, 2018; Tylka & Wood-Barcalow, 2015). Thus, interventions in negative BI would not necessarily promote positive BI (e.g., an individual with high levels of body appreciation can still experience body dissatisfaction) (Tiggemann & McCourt, 2013; Tylka & Wood-Barcalow, 2015).

Positive BI is characterized by the acceptance, appreciation, and respect for one’s body (Tylka, 2013). More specifically, according to Avalos et al. (2005), positive BI has four components: (1) favorable opinions about the body; (2) acceptance of the body with its imperfections, regardless of weight or body shape; (3) respect for the body by attending to its needs and engaging in healthy behaviors; and (4) protecting the body by rejecting unrealistic BIs portrayed in the media (e.g., positive media information is internalized, whereas negative media information is denied or reformulated).

Several studies state that positive BI is associated with healthy behaviors (Andrew et al., 2013; Gillen, 2015). According to Avalos et al. (2005), developing positive feelings towards the body can result in increased psychological well-being. Hence, positive BI is associated with lower development of ED symptoms (Wood-Barcalow et al., 2010) through its (1) direct influence on psychological well-being (Avalos et al., 2015); (2) indirect influence on reducing the impact of contextual influences (e.g., appearance-centered media) (Swami et al., 2008); and (3) promotion of protective cognitive styles (e.g., rejecting messages of criticism regarding one’s weight or interpreting ambiguous appearance-related messages as positive ones) and, as a result, higher resistance to the effects of appearance-centered media (Halliwell & Diedrichs, 2012).

In the past few years, mainly from the field of Positive Psychology, acceptance and appreciation of the body have been promoted as therapeutic targets for building a more positive BI. Programs designed to encourage body acceptance (e.g., not worrying about or exhibiting vanity about one’s appearance, rejecting socio-cultural ideals of beauty) can be more effective than programs that do not focus on this component (Stice et al., 2007). In addition, body appreciation ‒which implies an attitude of kindness, respect, and gratitude toward one’s bodily characteristics, functions, and physical condition‒ has been identified as a key protective factor of positive BI in young women (Wood-Barcalow et al., 2010). It promotes body acceptance by rejecting unrealistic ideals of beauty and enhancing individual psychological well-being by engaging in healthy behaviors (Avalos et al., 2005). In addition, body appreciation has been negatively related to risk factors associated with ED, such as body shame, body surveillance, and drive for thinness (Avalos et al., 2005).

In conclusion, positive BI stands out as a key dimension in BI that should be considered in the prevention and intervention of ED. Increasing positive BI by promoting body appreciation and recognition of one’s body needs goes beyond decreasing negative BI (Tylka, 2015). Promoting positive BI may have effective long-lasting effects and counteract the experience of disconnection from one’s body (i.e., disembodiment) (Tylka & Wood-Barcalow, 2015). Therefore, focusing on positive BI may help prevent BID intervention in individuals with ED (Piran, 2015; Tylka & Piran, 2019) by developing acceptance and respect towards their body (Avalos et al., 2005). However, more studies are needed to identify factors that enhance positive BI.

## Embodiment: considering the way we inhabit our body as a protective factor of positive BI

As noted above, BI is not an easily defined concept. Cash (2004) defined BI as a multifaceted psychological experience of embodying a body that involves self-perceptions, attitudes, thoughts, beliefs, feelings, and behaviors. Despite this complexity, the concept of embodiment has hardly been included in explanatory theories of BI. Nevertheless, as the latest research suggests that difficulty in embodying one’s body could contribute to the explanation of BID in ED.

According to her developmental theory of Embodiment (Piran, 2016), which integrates Buddhist psychology and mindfulness, the experience of positive embodiment includes five processes: (1) positive connection with the body, manifested by feeling comfortable and “at home” when embodying one’s body and interacting with the world from it; (2) experience of agency and functionality of one’s body (e.g., physical ability or body functions); (3) perception and awareness of bodily needs (e.g., hunger or sexual desire); (4) self-care in response to perceived internal needs (e.g., resting when tired or eating when hungry); and (5) embodying or “inhabiting” one’s body in the first person (as opposed to an objective or third-person perspective).

Although the concept of embodiment and positive BI dimensions may overlap (Menzel & Levine, 2011; Tylka, 2019) due to their focus on a positive connection with the body (Tylka & Piran, 2019), both constructs are different (Cook-Cottone, 2016). Developing a positive BI comes hand-in-hand with having a healthy, embodied awareness of internal and external aspects of self (Cook-Cottone, 2015). Positive embodiment promotes the growth of positive BI, as it involves a constructive connection with one’s body, which leads to caring for it with acceptance and non-judgment (Cook-Cottone, 2015; Piran, 2015), simultaneously encompassing all processes of the developmental theory of embodiment (Piran, 2016). In this regard, positive embodiment has been associated with mindfulness practice and, more specifically, the practice of self-compassion (Cook-Cottone, 2006, 2015; Tylka, 2012). Mindfulness practice has shown positive outcomes for variables that are negatively correlated with positive embodiment, such as body shame (Goldsmith et al., 2014; Woods & Proeve, 2014) and self-objectification (Cox et al., 2016).

In contrast, disembodiment implies the interruption of the connection with the body (the way it feels as well as its functions) (Tylka & Wood-Barcalow, 2015), which leads the person to perceive the body from an observer’s perspective (i.e., experience the body from a third-person perspective) (Menzel & Levine, 2011). Disembodiment has been positively associated with a lack of interoceptive awareness and a sense of disconnection from one’s body (Piran, 2015, 2016). According to Piran (2016), the lack of connection with the body could constitute an avoidance strategy that emerges in situations of discomfort where others can observe the body. Therefore, disembodiment, or the experience of adopting an observer’s perspective of one’s body by being an “object for others”, has been suggested as an altered mechanism in ED, a risk factor for negative BI. In this regard, disembodiment seems to be closely related to the concept of self-objectification. Self-objectification refers to the perception of oneself in the third person: the person perceives him/herself as an object that others evaluate based on physical appearance rather than on the body’s functionality or psychological qualities (Fredrickson & Roberts, 1997). Bodily self-objectification has been associated with increased body shame and decreased interoceptive awareness (Ainley et al., 2013), and it has been identified as an obstacle to body appreciation (Augustus-Horvath & Tylka, 2011). On the contrary, positive embodiment has been associated with less objectified body consciousness (Avalos et al., 2005; Menzel et al., 2011).

Lastly, another recent research area in the field of disembodiment focuses on studying mechanisms that underlie the experience of disconnection from the body in the ED patients. In recent years, research on the induction of perceptual illusions of ownership ‒mainly of a rubber hand (Botvinick & Cohen, 1998) or a full-body using visuo-tactile stimulation (Keizer et al., 2016)‒ has been carried out to induce the sense of embodiment with a false limb or a virtual avatar. This research area promotes the study of the basic components of embodiment (i.e., ownership, agency, and location of the body) in the disturbance of body representation. Findings indicate that patients with ED who show interoceptive deficits and self-objectification (Eshkevari et al., 2012; Herbert, 2020; Schaefer & Thompson, 2018), are more likely to detach (or experience disembodiment) from their body and embody another body or part of the body (e.g., a rubber hand) (Eshkevari et al., 2012, 2014; Keizer et al., 2014). That is, the fact of experiencing greater capability of embodying any other body, different from its own body, constitutes a sign of disembodiment in individuals with ED. This malleability of the bodily self persists even after ED recovery (Eshkevari et al., 2014). Therefore, a deeper understanding of the basic components of embodiment could promote long-lasting changes in the key mechanisms of BID by adjusting distorted body representations. For instance, the induction of bodily illusions by embodying a body that is thinner than one’s own results in lower body overestimation in women with AN (Keizer et al., 2016; Serino et al., 2019), as well as higher body satisfaction (Preston & Ehrsson, 2014; van der Hoort et al., 2011). Thus, induction of perceptual illusions that aim to manipulate the individual’s perception of the body -by making it thinner or fatter- is a promising alternative in the assessment and intervention of BID.

In conclusion, embodiment ‒or the way we inhabit or embody our body and the connection we establish with it‒ could be associated with our level of positive or negative BI. Therefore, assessing the experience of positive embodiment (or disembodiment) could contribute to a more comprehensive understanding of BI.

## Body shame: A risk factor associated with disembodiment and negative BI

Body shame is an emotion that is increasingly being addressed in recent studies of BID in ED (Cesare et al., 2016, Duarte et al., 2016, Mustapic et al., 2015, 2016). According to Gilbert (Gilbert, 2003; Gilbert & Miles, 2002), shame is a painful and self-conscious emotion that arises during the process of social competition as a warning sign that certain personal characteristics, attributes, or behaviors may be perceived as undesirable and, consequently, be judged negatively by others. The concept of shame has been divided into two dimensions: external shame and internal shame (Duarte et al., 2015; Gilbert, 2003). On the one hand, external shame arises when the individual perceives that s/he could be judged negatively by others (Gilbert & Miles, 2014; Tangney & Dearing, 2002). On the other hand, internal shame arises when the individual internalizes the negative judgment of others and, therefore, becomes her/his own judge (Gilbert, 2003).

More specifically, body shame has been studied within the affective dimension of negative BI (Menzel et al., 2011). It refers to a painful emotion that consists of cognitive, behavioral, affective, and social components related to appearance and body-related functions (Gilbert, 2003). The experience of body shame has mainly been associated with two theories that have attempted to explain the development and maintenance of ED symptoms.

On the one hand, the social comparison theory (Festinger, 1954) states that individuals, mainly women, tend to compare themselves with people from their social context. An unfavorable evaluation, experienced as inferiority, results in increased negative affect and reduced self-esteem. In this regard, evidence shows that social comparison has a negative impact on the level of body satisfaction (Myers & Crowther, 2009) because self-surveillance ‒or the act of observing oneself‒ is directly associated with appearance anxiety (Fredrickson & Roberts, 1997). Similarly, the anxiety experienced in exposure tasks using images of thin bodies has been shown to increase body dissatisfaction through the process of social comparison (Friederich et al., 2007). Therefore, this theory suggests that increased body dissatisfaction may be related to a higher tendency to observe anxiety-inducing body parts (Jansen et al., 2005).

On the other hand, the self-objectification theory posits that body shame arises from comparing one’s body to an internalized socio-cultural ideal (Fredrickson & Roberts, 1997). In other words, according to this theory, self-objectification has its origins in the internalization of the ideal of socio-cultural beauty, which entails the constant tendency to self-monitor the body and observe it from a third-person perspective. This process of self-monitoring and self-objectification leads to increased body shame, greater appearance anxiety, poor interoceptive awareness, increased negative affect (Miner-Rubino et al., 2002), and increased depressive symptoms (Muehlenkamp & Saris-Baglama, 2002; Szymanski & Henning, 2007). A negative self-evaluation in this context leads individuals to perceive themselves as inferior, unattractive, or unwanted (Duarte et al., 2015; Gilbert & Miles, 2014).

Both theories coincide in that a negative evaluation of one’s physical appearance resulting from social comparison leads to increased body shame (Cook-Cottone et al., 2008). Body shame is one of the most frequent consequences of the internalization of the Western body ideal (Lamont, 2019). In addition, body shame is one of the most common emotional states associated with negative BI in ED (Goss & Gilbert, 2014; Hayaki et al., 2002; Pinto-Gouveia et al., 2014), and it can be found in both clinical and non-clinical populations (Dakanalis et al., 2014; Doran & Lewis, 2011).

Furthermore, body shame is an emotion associated with the experience of disembodiment or disconnection from one’s body (Piran, 2016; Piran & Neumark-Sztainer, 2020). Therefore, it is important to identify strategies to reduce the experience of body shame. In this regard, self-compassion is emerging as a variable that protects against body shame and improves women’s BI (Halliwell, 2015). Some findings show that individuals with higher self-compassion levels have lower levels of body shame (Breines et al., 2014; Ferreira et al., 2019; Liss & Erchull, 2015). Hence, practice of self-compassion could constitute an intervention strategy to enhance positive embodiment ‒or a better way to inhabit or interact with one’s body‒.

## Self-compassion: A protective factor that promotes positive embodiment and positive BI?

Self-compassion, a concept derived from Buddhist psychology, involves an openness to perceiving one’s suffering as part of the human experience, without avoiding it or distancing oneself from it, and the desire to alleviate it with kindness and without judgment (Neff, 2003). The self-compassion construct consists of three main components (Neff, 2003): (1) mindfulness (vs over-identification), defined as the ability to observe thoughts and feelings, including body-related ones, without judgment or over-identification with them; (2) common humanity (vs isolation), defined as the ability to understand and identify one’s life experience as human and feel connected to others by identifying the experience as common (e.g., worrying about weight or not fulfilling the ideal of beauty); (3) self-kindness (vs self-criticism), defined as the ability to understand and be kind to oneself, take care of oneself, and accept one’s mistakes (e.g., being understanding when gaining weight).

Some evidence shows that self-compassion is a predictor of positive affect and happiness (Neff et al., 2007; Neff & Vonk, 2009). In a recent meta-analysis, self-compassion was identified as an adaptive emotional regulation strategy (Turk & Waller, 2020) associated with alleviating shame and self-criticism (Gilbert, 2010; Leary et al., 2007; Neff, 2003; Neff & Vonk, 2009). More specifically, the self-kindness component would prevent negative self-evaluations involving shame, whereas the mindfulness component would prevent generalizing errors to the whole self through the ability to maintain thoughts and feelings without over-identifying with them (e.g., the person can regard a mistake made as something transitory, without over-identifying with it) (Neff, 2003). Furthermore, the self-kindness component has been associated with understanding oneself during situations of stress and danger (Neff, 2003). Therefore, in stressful situations related to BI (e.g., viewing advertisements that include bodies that meet the ideal of beauty), an individual with higher levels of self-compassion will be better able to counteract the discomfort caused by these situations (e.g., less self-criticism related to body size and weight) (Webb et al., 2014). In the case of negative BI, the evidence suggests that self-compassion is associated with a decrease in concern about body size and weight, body shame, self-objectification, and the influence of internalizing the ideal of beauty (Braun et al., 2016; Ferreira et al., 2013; Wasylkiw et al., 2012).

In addition to its role in decreasing negative BI, self-compassion is considered a protective variable associated with the development and maintenance of positive BI (Braun et al., 2016; Neff, 2003; Siegel et al., 2020; Wasylkiw et al., 2012). The evidence suggests that there is a link between increased BI flexibility ‒defined as a compassionate response in accepting aversive body-related thoughts and feelings (Sandoz et al., 2013), increased acceptance of negative BI-related experiences (Daye et al., 2014; Kelly et al., 2014; Mosewich et al., 2011; Wasylkiw et al., 2012), and greater body appreciation (Ferreira et al., 2013). Therefore, high levels of self-compassion seem to contribute to lower negative BI and higher positive BI.

In this regard, Altman et al. (2017) developed the Body Compassion Scale to assess self-compassion related to one’s body. It combines the constructs of self-compassion (from Buddhist psychology) and BI (explained from the cognitive-behavioral approach). The scale has three dimensions: (1) defusion (e.g., “When I am frustrated with my body’s lack of ability to do something, I tend to feel alienated and isolated from others”); (2) common humanity (e.g., “When I am frustrated with some aspect of my appearance, I try to remind myself that most people feel this way all the time”); and (3) acceptance (e.g., “I accept my appearance as it is”). The scale is designed to assess individuals’ relationship with their body (e.g., presence of BID or positive BI) using an acceptance and mindfulness-based approach. Nonetheless, it is unclear whether body compassion (versus self-compassion) is a protective variable that explains more variance in the reduction of negative BI and the increase in positive BI, and whether body compassion (versus self-compassion) should have a more significant role in the assessment and treatment of BID.

Self-compassion focused interventions could contribute to increasing the connection with the body and decreasing self-objectification (Piran, 2015). These interventions try to modify individuals’ relationships with their appearance by fostering acceptance and appreciation of body size and weight, with the ultimate goal of promoting positive embodiment (vs disembodiment). They are aimed at promoting both body appreciation and self-care, buffering the tendency to compare oneself with others or with certain ideals (Avalos et al., 2005). The studies by Albertson et al. (2015) and Toole and Craighead (2016) analyzed the effectiveness of online interventions based on self-compassion in samples of undergraduate female students with high negative BI concerns. The results showed that the intervention programs were effective in increasing body appreciation and decreasing body shame and body dissatisfaction, among other effects. Similarly, self-compassionate letter writing is an effective intervention to promote treatment-seeking motivation in patients with anorexia nervosa (Kelly & Waring, 2018) and improve body satisfaction in undergraduate women (Stern & Engeln, 2018). Consequently, we can determine that the practice of self-compassion seems to be a promising area of intervention, not only for decreasing negative BI, but also for enhancing positive BI.

## Conclusions and future research directions in the study of BI

We carried out a narrative review of several protective and risk factors related to positive and negative BI (i.e., positive embodiment/disembodiment, body shame, and self-compassion) in order to understand this construct from a comprehensive perspective. We think this perspective should be taken into consideration in the assessment and intervention of BID in ED. Nonetheless, there are still many questions on this path that need to be clarified.

First, evidence points out the need to consider the positive (and not only the negative) dimension of BI for a comprehensive understanding of BID in patients with ED. To this end, additional and independent research on each of the specific components of positive BI (e.g., appreciation of body appearance or body functionality) is required in order to: (1) develop specific instruments (for both trait and state positive BI); and (2) integrate the BI positive components (e.g., body appreciation) into theoretical models that can explain the associations between these variables and the negative BI variables (e.g., body dissatisfaction).

Second, this review also highlights a long-neglected issue in the assessment and treatment of BI: the experience of embodiment. Although this aspect is included in some of the well-known definitions of BI, such as Cash’s (2004), it has not been thoroughly studied. This research field may provide novel experimental paradigms to explore the underlying mechanisms of positive embodiment (e.g., self-compassion) in patients with ED. More specifically, deeper understanding of how embodiment is developed may help improve prevention and intervention programs for BID by enhancing the psychological processes responsible for positive connections to one’s own body.

In addition, following the positive embodiment model incorporated in the developmental theory of Embodiment (Piran, 2015), discussed in this paper, there is a need (1) to conduct more studies related to activities that promote positive embodiment (e.g., yoga or exercise) and have benefits for body awareness or the experience of self-objectification, among others; (2) to develop instruments to delimit the different dimensions of positive embodiment; (3) to explore how risk factors (e.g., disembodiment) interact with protective factors of BI (e.g., positive embodiment) (Piran, 2016); and, finally, (4) to define underlying mechanisms of the association between the concepts of positive embodiment and self-compassion, as well as disembodiment and body shame.

Third, in recent years, the need to integrate body shame assessment as part of the impairment in the BID affective dimension has been highlighted. This variable appears to be associated with disembodiment, and through this interaction, body shame could lead to negative BI. Additionally, along with body shame, this review indicates the relevance of self-compassion because it can play a relevant role in fostering positive BI by cultivating positive embodiment. Therefore, it is necessary (1) to identify the mechanisms of action of self-compassionate practice and its effect on the decrease in body shame, as well as the promotion of positive BI, (2) to establish the role of the constructs of body self-compassion versus self-compassion in promoting positive embodiment and healthy BI, and finally, (3) to design effective interventions that integrate self-compassion to reduce body shame, increase positive embodiment, and consequently, increase positive BI and reduce negative BI.

Inclusion of these protective and risk factors in theoretical BI models has the potential to provide a comprehensive perspective of this complex concept and may allow using strategies and instruments to improve BI assessment, prevention, and treatment in patients with ED. Hence, more studies are required to establish the protective role of positive embodiment and self-compassion in the development, maintenance, and relapse of ED. Moreover, a shift in future study designs is needed to better understand the variables described in this review: greater diversity in the samples and the implementation of longitudinal studies. It is necessary to strive for greater heterogeneity among the participants because most of the published studies have been conducted on young, white, heterosexually oriented adult women with significant concerns about BI and no physical disabilities (Atkinson & Wade, 2016; Toole & Craighead, 2016). To develop effective BID-related interventions in ED, full understanding of the BI construct is required considering positive BI (e.g., body functionality, body flexibility) and negative BI (e.g., body disgust, “feeling fat”) dimensions. In this regard, it is essential to explore different populations to capture all risk and protective factors involved in BID. Therefore, future studies should include a representative sample of diverse cultural groups, different age groups (especially children and the elderly), and the male population.

In addition to increasing sample diversity, a thorough examination of life transition periods (e.g., adolescence, pregnancy, or menopause) is required due to their impact on BI development and modification (Piran, 2015). Studying the impact of time on the different components of BI and the embodiment experience could lead to the development of specific interventions that may address specific protective and risk factors during each period. For example, the prevalence of negative BI in adolescents indicates the relevance of prevention programs for this age group by identifying specific variables that would facilitate the promotion of healthy BI, such as body acceptance. Likewise, there is a need to conduct longitudinal studies in order to examine the causal relationships between the aforementioned variables. The results of these studies could be incorporated into the theoretical models of the psychological processes involved in the development and maintenance of positive BI and prevention of BID in ED. Moreover, dismantling studies (e.g., Roehrig et al., 2006) would help to determine the role of the components of the BI dimensions and their relationships, in addition to designing interventions with specific components for healthy BI development.

In conclusion, this review emphasizes the importance of considering new protective and risk factors -and the links they maintain with each other- of BI conceptualization, to continue to advance in its understanding. Inclusion of the positive dimension of BI, and considering positive embodiment and self-compassion as protective factors -opposed to the disconnection from our body and body shame- will allow us to reach a deeper and more comprehensive understanding of the BI construct. This perspective may lead to a more suitable approach for researching and developing future prevention and intervention programs focusing not only on reducing negative BI (through decreasing body shame and disembodiment), but also on positive BI connection with one’s own body (through increasing self-compassion and positive embodiment).

## References

[1] Ainley, V., Maister, L., Brokfeld, J., Farmer, H., & Tsakiris, M. (2013). More of myself: Manipulating interoceptive awareness by heightened attention to bodily and narrative aspects of the self. Consciousness and Cognition, 22(4), 1231–1238. DOI: 10.1016/j.concog.2013.08.004 24021852

[2] Albertson, E. R., Neff, K. D., & Dill-shackleford, K. E. (2015). Self-Compassion and Body Dissatisfaction in Women: A Randomized Controlled Trial of a Brief Meditation Intervention. Mindfulness, 6(3), 444–454. DOI: 10.1007/s12671-014-0277-3

[3] Alleva, J. M., Sheeran, P., Webb, T. L., Martijn, C., & Miles, E. (2015). A meta-analytic review of stand-alone interventions to improve body image. PLoS One, 10(9), e0139177. DOI: 10.1371/journal.pone.0139177 26418470 PMC4587797

[4] Altman, J. K., Linfield, K., Salmon, P. G., & Beacham, A. O. (2017). The body compassion scale: Development and initial validation. Journal of Health Psychology, 25(4), 439–449. DOI: 10.1177/1359105317718924 28810491

[5] Andrew, R., Tiggemann, M., & Clark, L. (2013). Self-compassion and positive body image: a role for social comparison? Journal of Eating Disorders, 1(S1), O54. DOI: 10.1186/2050-2974-1-S1-O54

[6] Atkinson, M. J., & Wade, T. D. (2016). Does mindfulness have potential in eating disorders prevention? A preliminary controlled trial with young adult women. Early Intervention in Psychiatry, 10(3), 234–245. DOI: 10.1111/eip.12160 24894735

[7] Augustus-Horvath, C. L., & Tylka, T. L. (2011). The Acceptance Model of Intuitive Eating: A Comparison of Women in Emerging Adulthood, Early Adulthood, and Middle Adulthood. Journal of Counseling Psychology, 58(1), 110–125. DOI: 10.1037/a0022129 21244144

[8] Avalos, L., Tylka, T., & Wood-Barcalow, N. (2005). The Body Appreciation Scale: Development and psychometric evaluation. Body Image, 2(3), 285–297. DOI: 10.1016/j.bodyim.2005.06.002 18089195

[9] Bachner-Melman, R., Zohar, A. H., & Ebstein, R. P. (2006). An Examination of Cognitive Versus Behavioral Components of Recovery From Anorexia Nervosa. The Journal of Nervous and Mental Disease, 194(9), 697–703. DOI: 10.1097/01.nmd.0000235795.51683.99 16971822

[10] Botvinick, M., & Cohen, J. (1998). Rubber hands ‘feel’ touch that eyes see. Nature, 391(6669), 756. DOI: 10.1038/35784 9486643

[11] Braun, T. D., Park, C. L., & Gorin, A. (2016). Self-compassion, body image, and disordered eating: A review of the literature. Body Image, 17, 117–131. DOI: 10.1016/j.bodyim.2016.03.003 27038782

[12] Breines, J. G., Thoma, M. V., Gianferante, D., Hanlin, L., Chen, X., & Rohleder, N. (2014). Self-compassion as a predictor of interleukin-6 response to acute psychosocial stress. Brain, Behavior, and Immunity, 37, 109–114. DOI: 10.1016/j.bbi.2013.11.006 PMC4311753 24239953

[13] Brown, T. A., Shott, M. E., & Frank, G. K. (2021). Body size overestimation in anorexia nervosa: Contributions of cognitive, affective, tactile and visual information. Psychiatry Research, 297, 113705. DOI: 10.1016/j.psychres.2021.113705 33472094 PMC11537156

[14] Bulik, C. M., Sullivan, P. F., Tozzi, F., Furberg, H., Lichtenstein, P., & Pedersen, N. L. (2006). Prevalence, Heritability, and Prospective Risk Factors for Anorexia Nervosa. Archives of General Psychiatry, 63(3), 305–312. DOI: 10.1001/archpsyc.63.3.305 16520436

[15] Carter, J. C., Blackmore, E., Sutandar-Pinnock, K., & Woodside, D. B. (2004). Relapse in anorexia nervosa: a survival analysis. Psychological Medicine, 34(4), 671–679. DOI: 10.1017/S0033291703001168 15099421

[16] Cash, T. F. (2002). The situational inventory of body-image dysphoria: Psychometric evidence and development of a short form. International Journal of Eating Disorders, 32(3), 362–366. DOI: 10.1002/eat.10100 12210651

[17] Cash, T. F. (2004). Body image: past, present, and future. Body Image, 1(1), 1–5. DOI: 10.1016/S1740-1445(03)00011-1 18089136

[18] Cash, T. F., & Deagle, E. A. (1997). The nature and extent of body-image disturbances in anorexia nervosa and bulimia nervosa: A meta-analysis. International Journal of Eating Disorders, 22(2), 107–126. DOI: 10.1002/(SICI)1098-108X(199709)22:2<107::AID-EAT1>3.0.CO;2-J 9261648

[19] Cesare, C., Francesco, P., Valentino, Z., Barbara, D., Olivia, R., Gianluca, C., Patrizia, T., & Enrico, M. (2016). Shame proneness and eating disorders: a comparison between clinical and non-clinical samples. Eating and Weight Disorders – Studies on Anorexia, Bulimia and Obesity, 21(4), 701–707. DOI: 10.1007/s40519-016-0328-y 27704341

[20] Cook-Cottone, C. (2006). The attuned representation model for the primary prevention of eating disorders: An overview for school psychologists. Psychology in the Schools, 43(2), 223–230. DOI: 10.1002/pits.20139

[21] Cook-Cottone, C. (2015). Incorporating positive body image into the treatment of eating disorders: A model for attunement and mindful self-care. Body Image, 14, 158–167. DOI: 10.1016/j.bodyim.2015.03.004 25886712

[22] Cook-Cottone, C. (2016). Embodied self-regulation and mindful self-care in the prevention of eating disorders. Eating disorders, 24(1), 98–105. DOI: 10.1080/10640266.2015.1118954 26652657

[23] Cook-Cottone, C. (2018). Mindful Self-Care and Positive Body Image: Mindfulness, Yoga, and Actionable Tools for Positive Embodiment. In E. Daniels, M. Gillen & C. Markey (Eds.), Body Positive: Understanding and Improving Body Image in Science and Practice (pp. 135–159). Cambridge University Press. DOI: 10.1017/9781108297653.007

[24] Cook-Cottone, C., Beck, M., & Kane, L. (2008). Manualized-group treatment of eating disorders: Attunement in mind, body, and relationship (AMBR). Journal for Specialists in Group Work, 33(1), 61–83. DOI: 10.1080/01933920701798570

[25] Cornelissen, K. K., Bester, A., Cairns, P., Tovée, M. J., & Cornelissen, P. L. (2015). The influence of personal BMI on body size estimations and sensitivity to body size change in anorexia spectrum disorders. Body Image, 13, 75–85. DOI: 10.1016/j.bodyim.2015.01.001 25697956

[26] Cornelissen, P. L., Johns, A., & Tovée, M. J. (2013). Body size over-estimation in women with anorexia nervosa is not qualitatively different from female controls. Body Image, 10(1), 103–111. DOI: 10.1016/j.bodyim.2012.09.003 23102545

[27] Cox, A. E., Ullrich-French, S., Cole, A. N., & D’Hondt-Taylor, M. (2016). The role of state mindfulness during yoga in predicting self-objectification and reasons for exercise. Psychology of Sport and Exercise, 22, 321–327. DOI: 10.1016/j.psychsport.2015.10.001

[28] Dakanalis, A., Gaudio, S., Serino, S., Clerici, M., Carrà, G., & Riva, G. (2016). Body-image distortion in anorexia nervosa. Nature Reviews Disease Primers, 2(1), 1–2. DOI: 10.1038/nrdp.2016.26

[29] Dakanalis, A., Timko, C. A., Carrà, G., Clerici, M., Zanetti, M. A., Riva, G., & Caccialanza, R. (2014). Testing the original and the extended dual-pathway model of lack of control over eating in adolescent girls. A two-year longitudinal study. Appetite, 82, 180–193. DOI: 10.1016/j.appet.2014.07.022 25058649

[30] Daye, C. A., Webb, J. B., & Jafari, N. (2014). Exploring self-compassion as a refuge against recalling the body-related shaming of caregiver eating messages on dimensions of objectified body consciousness in college women. Body Image, 11(4), 547–556. DOI: 10.1016/j.bodyim.2014.08.001 25195124

[31] Degortes, D., Santonastaso, P., & Favaro, A. (2018). Body Image Disturbances in Bulimia Nervosa. In M. Cuzzolaro, & S. Fassino (Eds.), Body Image, Eating, and Weight (pp. 127–140). Springer International Publishing. DOI: 10.1007/978-3-319-90817-5_9

[32] Doran, J., & Lewis, C. A. (2011). Components of Shame and Eating Disturbance Among Clinical and Non-clinical Populations. European Eating Disorders Review, 20(4), 265–270. DOI: 10.1002/erv.1142 21800398

[33] Duarte, C., Ferreira, C., & Pinto-Gouveia, J. (2016). At the core of eating disorders: Overvaluation, social rank, self-criticism and shame in anorexia, bulimia and binge eating disorder. Comprehensive Psychiatry, 66, 123–131. DOI: 10.1016/j.comppsych.2016.01.003 26995245

[34] Duarte, C., Pinto-Gouveia, J., Ferreira, C., & Batista, D. (2015). Body Image as a Source of Shame: A New Measure for the Assessment of the Multifaceted Nature of Body Image Shame. Clinical Psychology and Psychotherapy, 22(6), 656–666. DOI: 10.1002/cpp.1925 25316416

[35] Engel, M. M., & Keizer, A. (2017). Body representation disturbances in visual perception and affordance perception persist in eating disorder patients after completing treatment. Scientific reports, 7(1), 1–9. DOI: 10.1038/s41598-017-16362-w 29170439 PMC5701063

[36] Eshkevari, E., Rieger, E., Longo, M. R., Haggard, P., & Treasure, J. (2012). Increased plasticity of the bodily self in eating disorders. Psychological Medicine, 42(4), 819–828. DOI: 10.1017/S0033291711002091 22017964

[37] Eshkevari, E., Rieger, E., Longo, M., Haggard, P., & Treasure, J. (2014). Persistent body image disturbance following recovery from eating disorders. International Journal of Eating Disorders, 47(4), 400–409. DOI: 10.1002/eat.22219 24243423

[38] Ferreira, C., Dias, B., & Oliveira, S. (2019). Behind women’s body image-focused shame: Exploring the role of fears of compassion and self-criticism. Eating Behaviors, 32, 12–17. DOI: 10.1016/j.eatbeh.2018.11.002 30471481

[39] Ferreira, C., Pinto-Gouveia, J., & Duarte, C. (2013). Self-compassion in the face of shame and body image dissatisfaction: Implications for eating disorders. Eating Behaviors, 14(2), 207–210. DOI: 10.1016/j.eatbeh.2013.01.005 23557822

[40] Festinger, L. (1954). A Theory of Social Comparison Processes. Human Relations, 7(2), 117–140. DOI: 10.1177/001872675400700202

[41] Fredrickson, B. L., & Roberts, T. A. (1997). Toward understanding women’s lived experiences and mental health risks. Psychology of Women Quarterly, 21(2), 173–206. DOI: 10.1111/j.1471-6402.1997.tb00108.x

[42] Friederich, H. C., Uher, R., Brooks, S., Giampietro, V., Brammer, M., Williams, S. C. R., Herzog, W., Treasure, J., & Campbell, I. C. (2007). I’m not as slim as that girl: Neural bases of body shape self-comparison to media images. NeuroImage, 37(2), 674–681. DOI: 10.1016/j.neuroimage.2007.05.039 17604649

[43] Gardner, R. M. (1996). Methodological issues in assessment of the perceptual component of body image disturbance. British Journal of Psychology, 87(2), 327–337. DOI: 10.1111/j.2044-8295.1996.tb02593.x 8673361

[44] Gardner, R. M., & Bokenkamp, E. D. (1996). The role of sensory and nonsensory factors in body size estimations of eating disorder subjects. Journal of Clinical Psychology, 52(1), 3–15. DOI: 10.1002/(SICI)1097-4679(199601)52:1<3::AID-JCLP1>3.0.CO;2-X 8682909

[45] Gardner, R. M., & Brown, D. L. (2014). Body size estimation in anorexia nervosa: A brief review of findings from 2003 through 2013. Psychiatry Research, 219(3), 407–410. DOI: 10.1016/j.psychres.2014.06.029 25023364

[46] Garner, D. M., & Garfinkel, P. E. (1982). Body Image in Anorexia Nervosa: Measurement, Theory and Clinical Implications. The International Journal of Psychiatry in Medicine, 11(3), 263–284. DOI: 10.2190/R55Q-2U6T-LAM7-RQR7 7309395

[47] Gilbert, P. (2003). Evolution, Social Roles, and the Differences in Shame and Guilt. Social Research, 70(4), 1205–1230.

[48] Gilbert, P. (2010). An Introduction to Compassion Focused Therapy in Cognitive Behavior Therapy. International Journal of Cognitive Therapy, 3(2), 97–112. DOI: 10.1521/ijct.2010.3.2.97

[49] Gilbert, P., & Miles, J. (Eds.) (2014). Body shame: Conceptualisation, research and treatment. Routledge. DOI: 10.4324/9781315820255

[50] Gillen, M. M. (2015). Associations between positive body image and indicators of men’s and women’s mental and physical health. Body Image, 13, 67–74. DOI: 10.1016/j.bodyim.2015.01.002 25682474

[51] Glashouwer, K. A., van der Veer, R. M. L., Adipatria, F., de Jong, P. J., & Vocks, S. (2019). The role of body image disturbance in the onset, maintenance, and relapse of anorexia nervosa: A systematic review. Clinical Psychology Review, 74. DOI: 10.1016/j.cpr.2019.101771 31751876

[52] Goldsmith, R. E., Gerhart, J. I., Chesney, S. A., Burns, J. W., Kleinman, B., & Hood, M. M. (2014). Mindfulness-Based Stress Reduction for Posttraumatic Stress Symptoms. Journal of Evidence-Based Complementary & Alternative Medicine, 19(4), 227–234. DOI: 10.1177/2156587214533703 24812075

[53] Goss, K., & Gilbert, P. (2014). Eating disorders, shame and pride: A cognitive-behavioural functional analysis. In P. Gilbert & J. Miles (Eds.), Body Shame: Conceptualisation, Research and Treatment. Routledge.

[54] Grogan, S. (2016) Body image: understanding body dissatisfaction in men, women and children. (3rd ed). Taylor & Francis.

[55] Hagman, J., Gardner, R. M., Brown, D. L., Gralla, J., Fier, J. M., & Frank, G. K. W. (2015). Body size overestimation and its association with body mass index, body dissatisfaction, and drive for thinness in anorexia nervosa. Eating and Weight Disorders – Studies on Anorexia, Bulimia and Obesity, 20(4), 449–455. DOI: 10.1007/s40519-015-0193-0 25929983

[56] Halliwell, E. (2015). Future directions for positive body image research. Body image, 14, 177–189. DOI: 10.1016/j.bodyim.2015.03.003 25861909

[57] Halliwell, E., & Diedrichs, P. C. (2012). Influence of the media. In N. Rumsey & D. Harcourt (Eds.), The Oxford handbook of the psychology of appearance (pp. 217–238). Oxford University Press. DOI: 10.1093/oxfordhb/9780199580521.013.0019

[58] Hayaki, J., Friedman, M. A., & Brownell, K. D. (2002). Shame and severity of bulimic symptoms. Eating Behaviors, 3(1), 73–83. DOI: 10.1016/S1471-0153(01)00046-0 15001021

[59] Herbert, B. M. (2020). Interoception and its role for eating, obesity, and eating disorders: Empirical findings and conceptual conclusions. European Journal of Health Psychology, 27(4), 188–205. DOI: 10.1027/2512-8442/a000062

[60] Homan, K. J., & Tylka, T. L. (2014). Appearance-based exercise motivation moderates the relationship between exercise frequency and positive body image. Body Image, 11, 101–108. DOI: 10.1016/j.bodyim.2014.01.003 24529336

[61] Hosseini, S., & Padhy, R. (2019). Body Image Distortion. In StatPearls [Internet]. https://europepmc.org/books/n/statpearls/article-18424/31536191

[62] Jansen, A., Nederkoorn, C., & Mulkens, S. (2005). Selective visual attention for ugly and beautiful body parts in eating disorders. Behaviour Research and Therapy, 43(2), 183–196. DOI: 10.1016/j.brat.2004.01.003 15629749

[63] Keizer, A., Aldegonda, M., Smeets, M., Christiaan, H., Van Den Hout, M., Klugkist, I., Van Elburg, A., & Postma, A. (2011). Tactile body image disturbance in anorexia nervosa. Psychiatry Research, 190(1), 115–120. DOI: 10.1016/j.psychres.2011.04.031 21621275

[64] Keizer, A., Smeets, M. A. M., Postma, A., van Elburg, A., & Dijkerman, H. C. (2014). Does the experience of ownership over a rubber hand change body size perception in anorexia nervosa patients? Neuropsychologia, 62, 26–37. DOI: 10.1016/j.neuropsychologia.2014.07.003 25050852

[65] Keizer, A., van Elburg, A., Helms, R., & Dijkerman, H. C. (2016). A Virtual Reality Full Body Illusion Improves Body Image Disturbance in Anorexia Nervosa. PLOS ONE, 11(10), e0163921. DOI: 10.1371/journal.pone.0163921 27711234 PMC5053411

[66] Kelly, A., & Waring, S. (2018). A feasibility study of a 2-week self-compassionate letter-writing intervention for nontreatment seeking individuals with typical and atypical anorexia nervosa. International Journal of Eating Disorders, 51(8), 1005–1009. DOI: 10.1002/eat.22930 30102787

[67] Kelly, A., Vimalakanthan, K., & Miller, K. (2014). Self-compassion moderates the relationship between body mass index and both eating disorder pathology and body image flexibility. Body Image, 11(4), 446–453. DOI: 10.1016/j.bodyim.2014.07.005 25113286

[68] Lamont, J. M. (2019). The Relationship of Mindfulness to Body Shame, Body Responsiveness, and Health Outcomes. Mindfulness, 10(4), 639–649. DOI: 10.1007/s12671-018-1020-2

[69] Leary, M. R., Tate, E. B., Adams, C. E., Batts Allen, A., & Hancock, J. (2007). Self-compassion and reactions to unpleasant self-relevant events: The implications of treating oneself kindly. Journal of Personality and Social Psychology, 92(5), 887–904. DOI: 10.1037/0022-3514.92.5.887 17484611

[70] Linardon, J. (2018). Rates of abstinence following psychological or behavioral treatments for binge-eating disorder: Meta-analysis. International Journal of Eating Disorders, 51(8), 785–797. DOI: 10.1002/eat.22897 30058074

[71] Linardon, J., & Wade, T. D. (2018). How many individuals achieve symptom abstinence following psychological treatments for bulimia nervosa? A meta-analytic review. International Journal of Eating Disorders, 51(4), 287–294. DOI: 10.1002/eat.22838 29417609

[72] Linardon, J., Wade, T. D., De La Piedad Garcia, X., & Brennan, L. (2017). The efficacy of cognitive-behavioral therapy for eating disorders: A systematic review and meta-analysis. Journal of Consulting and Clinical Psychology, 85(11), 1080–1094. DOI: 10.1037/ccp0000245 29083223

[73] Liss, M., & Erchull, M. J. (2015). Not hating what you see: Self-compassion may protect against negative mental health variables connected to self-objectification in college women. Body Image, 14, 5–12. DOI: 10.1016/j.bodyim.2015.02.006 25828840

[74] McCabe, M. P., Ricciardelli, L. A., Sitaram, G., & Mikhail, K. (2006). Accuracy of body size estimation: Role of biopsychosocial variables. Body Image, 3(2), 163–171. DOI: 10.1016/j.bodyim.2006.01.004 18089219

[75] Menzel, J., Krawczyk, R., & Thompson, J. K. (2011). Attitudinal assessment of body image for adolescents and adults. In T. F. Cash & L. Smolak (Eds.), Body image: A handbook of science, practice, and prevention. (2nd ed., pp. 154–169). The Guilford Press.

[76] Menzel, J., & Levine, M. (2011). Embodying experiences and the promotion of positive body image: The example of competitive athletics. In R. M. Calogero, S. Tantleff-Dunn & J. K. Thompson (Eds.), Self-objectification in women: Causes, consquences, and counteractions. (pp. 163–186). American Psychological Association. DOI: 10.1037/12304-008

[77] Miner-Rubino, K., Twenge, J., & Fredrickson, B. (2002). Trait Self-Objectification in Women: Affective and Personality Correlates. Journal of Research in Personality, 36(2), 147–172. DOI: 10.1006/jrpe.2001.2343

[78] Mitchison, D., Mond, J., Bussey, K., Griffiths, S., Trompeter, N., Lonergan, A., Pike, K. M., Murray, S. B., & Hay, P. (2020). DSM-5 full syndrome, other specified, and unspecified eating disorders in Australian adolescents: prevalence and clinical significance. Psychological Medicine, 50(6), 981–990. DOI: 10.1017/S0033291719000898 31043181

[79] Mölbert, S. C., Klein, L., Thaler, A., Mohler, B. J., Brozzo, C., Martus, P., Karnath, H. O., Zipfel, S., & Giel, K. E. (2017). Depictive and metric body size estimation in anorexia nervosa and bulimia nervosa: A systematic review and meta-analysis. Clinical Psychology Review, 57, 21–31. DOI: 10.1016/j.cpr.2017.08.005 28818670

[80] Mölbert, S. C., Thaler, A., Mohler, B. J., Streuber, S., Romero, J., Black, M. J., & Zipfel, S. (2018). Assessing body image in anorexia nervosa using biometric self-avatars in virtual reality: Attitudinal components rather than visual body size estimation are distorted. Psychological Medicine, 48(4), 642–653. DOI: 10.1017/S0033291717002008 28745268 PMC5964466

[81] Mora-Giral, M., Raich-Escursell, R. M., Segues, C. V., Torras-Clarasó, J., & Huon, G. (2004). Bulimia symptoms and risk factors in university students. Eating and Weight Disorders – Studies on Anorexia, Bulimia and Obesity, 9(3), 163–169. DOI: 10.1007/BF03325062 15656009

[82] Moscone, A. L., Amorim, M. A., Le Scanff, C., & Leconte, P. (2017). A model-driven approach to studying dissociations between body size mental representations in anorexia nervosa. Body Image, 20, 40–48. DOI: 10.1016/j.bodyim.2016.11.003 27912194

[83] Mosewich, A. D., Kowalski, K. C., Sabiston, C. M., Sedgwick, W. A., & Tracy, J. L. (2011). Self-Compassion: A Potential Resource for Young Women Athletes. Journal of Sport and Exercise Psychology, 33(1), 103–123. DOI: 10.1123/jsep.33.1.103 21451173

[84] Muehlenkamp, J. J., & Saris-Baglama, R. N. (2002). Self-objectification and its psychological outcomes for college women. Psychology of Women Quarterly, 26(4), 371–379. DOI: 10.1111/1471-6402.t01-1-00076

[85] Mustapic, J., Marcinko, D., & Vargek, P. (2015). Eating behaviours in adolescent girls: the role of body shame and body dissatisfaction. Eating and Weight Disorders-Studies on Anorexia, Bulimia and Obesity, 20(3), 329–335. DOI: 10.1007/s40519-015-0183-2 25701441

[86] Mustapic, J., Marcinko, D., & Vargek, P. (2016). Body Shame and Disordered Eating in Adolescents. Current Psychology, 36(3), 447–452. DOI: 10.1007/s12144-016-9433-3

[87] Myers, T. A., & Crowther, J. H. (2009). Social Comparison as a Predictor of Body Dissatisfaction: A Meta-Analytic Review. Journal of Abnormal Psychology, 118(4), 683–698. DOI: 10.1037/a0016763 19899839

[88] Neff, K. D. (2003). Self-Compassion Scale. Self and Identity, 2, 223–250. DOI: 10.1080/15298860309027

[89] Neff, K. D., & Vonk, R. (2009). Self-Compassion Versus Global Self-Esteem: Two Different Ways of Relating to Oneself. Journal of Personality, 77(1), 23–50. DOI: 10.1111/j.1467-6494.2008.00537.x 19076996

[90] Neff, K. D., Kirkpatrick, K. L., & Rude, S. S. (2007). Self-compassion and adaptive psychological functioning. Journal of Research in Personality, 41(1), 139–154. DOI: 10.1016/j.jrp.2006.03.004

[91] Pinto-Gouveia, J., Ferreira, C., & Duarte, C. (2014). Thinness in the Pursuit for Social Safeness: An Integrative Model of Social Rank Mentality to Explain Eating Psychopathology. Clinical Psychology & Psychotherapy, 21(2), 154–165. DOI: 10.1002/cpp.1820 23024036

[92] Piran, N. (2015). New possibilities in the prevention of eating disorders: The introduction of positive body image measures. Body Image, 14, 146–157. DOI: 10.1016/j.bodyim.2015.03.008 25886711

[93] Piran, N. (2016). Embodied possibilities and disruptions: The emergence of the Experience of Embodiment construct from qualitative studies with girls and women. Body Image, 18, 43–60. DOI: 10.1016/j.bodyim.2016.04.007 27236476

[94] Piran, N., & Neumark-Sztainer, D. (2020). Yoga and the experience of embodiment: a discussion of possible links. Eating Disorders, 28(4), 330–348. DOI: 10.1080/10640266.2019.1701350 31922924 PMC7347427

[95] Piran, N., & Teall, T. (2012). The developmental theory of embodiment. In G. L. McVey, M. P. Levine, N. Piran & H. B. Ferguson (Eds.), Preventing Eating-Related and Weight-Related Disorders: Collaborative Research, Advocacy, and Policy Change. (pp. 169–198). DOI: 10.4324/9781315159645-7

[96] Preston, C., & Ehrsson, H. H. (2014). Illusory Changes in Body Size Modulate Body Satisfaction in a Way That Is Related to Non-Clinical Eating Disorder Psychopathology. PLoS ONE, 9(1), e85773. DOI: 10.1371/journal.pone.0085773 24465698 PMC3897512

[97] Preston, C., & Ehrsson, H. H. (2016). Illusory obesity triggers body dissatisfaction responses in the insula and anterior cingulate cortex. Cerebral Cortex, 26(12), 4450–4460. DOI: 10.1093/cercor/bhw313 27733537 PMC5193143

[98] Riva, G., Gaudio, S., & Dakanalis, A. (2015). The Neuropsychology of Self-Objectification. European Psychologist, 20(1), 34–43. DOI: 10.1027/1016-9040/a000190

[99] Roehrig, M., Thompson, J. K., Brannick, M., & Van Den Berg, P. (2006). Dissonance-based eating disorder prevention program: A preliminary dismantling investigation. International Journal of Eating Disorders, 39(1), 1–10. DOI: 10.1002/eat.20217 16254869

[100] Sandoz, E. K., Wilson, K. G., Merwin, R. M., & Kate Kellum, K. (2013). Assessment of body image flexibility: The Body Image-Acceptance and Action Questionnaire. Journal of Contextual Behavioral Science, 2(1–2), 39–48. DOI: 10.1016/j.jcbs.2013.03.002

[101] Sattler, F. A., Eickmeyer, S., & Eisenkolb, J. (2019). Body image disturbance in children and adolescents with anorexia nervosa and bulimia nervosa: a systematic review. Eating and Weight Disorders – Studies on Anorexia, Bulimia and Obesity, 25, 857–865. DOI: 10.1007/s40519-019-00725-5 31177379

[102] Schaefer, L. M., & Thompson, J. K. (2018). Self-objectification and disordered eating: A meta-analysis. International Journal of Eating Disorders, 51(6), 483–502. DOI: 10.1002/eat.22854 PMC6002885 29517805

[103] Schilder, P. (1935). Psycho-analysis of space. International Journal of Psycho-Analysis, 16, 274–295.

[104] Serino, S., Polli, N., & Riva, G. (2019). From avatars to body swapping: The use of virtual reality for assessing and treating body-size distortion in individuals with anorexia. Journal of Clinical Psychology, 75(2), 313–322. DOI: 10.1002/jclp.22724 30552669 PMC6590458

[105] Siegel, J. A., Huellemann, K. L., Hillier, C. C., & Campbell, L. (2020). The protective role of self-compassion for women’s positive body image: an open replication and extension. Body Image, 32, 136–144. DOI: 10.1016/j.bodyim.2019.12.003 31887640

[106] Smolak, L., & Cash, T. (2011). Future challenges for body image science, practice, and prevention. In T. F. Cash & L. Smolak (Eds.), Body image: A handbook of science, practice, and prevention (pp. 471–478). Guilford Press.

[107] Smolak, L. (2012). Appearance in childhood and adolescence. In N. Rumsey & D. Harcourt (Eds.), The Oxford handbook of the psychology of appearance (pp. 123–144). OUP Oxford. DOI: 10.1093/oxfordhb/9780199580521.001.0001

[108] Stern, N. G., & Engeln, R. (2018). Self-Compassionate Writing Exercises Increase College Women’s Body Satisfaction. Psychology of Women Quarterly, 42(3), 326–341. DOI: 10.1177/0361684318773356

[109] Stice, E., & Shaw, H. E. (2002). Role of body dissatisfaction in the onset and maintenance of eating pathology. Journal of Psychosomatic Research, 53(5), 985–993. DOI: 10.1016/S0022-3999(02)00488-9 12445588

[110] Stice, E., Shaw, H., & Marti, C. N. (2007). A Meta-Analytic Review of Eating Disorder Prevention Programs: Encouraging Findings. Annual Review of Clinical Psychology, 3(1), 207–231. DOI: 10.1146/annurev.clinpsy.3.022806.091447 17716054

[111] Stice, E., & Whitenton, K. (2002). Risk factors for body dissatisfaction in adolescent girls: A longitudinal investigation. Developmental Psychology, 38(5), 669–678. DOI: 10.1037/0012-1649.38.5.669 12220046

[112] Swami, V., Hadji-Michael, M., & Furnham, A. (2008). Personality and individual difference correlates of positive body image. Body Image, 5(3), 322–325. DOI: 10.1016/j.bodyim.2008.03.007 18585107

[113] Szymanski, D. M., & Henning, S. L. (2007). The Role of Self-objectification in Women’s Depression: A Test of Objectification Theory. Sex Roles, 56(1–2), 45–53. DOI: 10.1007/s11199-006-9147-3

[114] Tangney, J., & Dearing, R. (2002). Emotions and social behavior. Shame and guilt. Guilford Press. DOI: 10.4135/9781412950664.n388

[115] Thompson, J. K., Coovert, M. D., & Stormer, S. M. (1999). Body image, social comparison, and eating disturbance: A covariance structure modeling investigation. International Journal of Eating Disorders, 26(1), 43–51. DOI: 10.1002/(SICI)1098-108X(199907)26:1<43::AID-EAT6>3.0.CO;2-R 10349583

[116] Tiggemann, M., & McCourt, A. (2013). Body appreciation in adult women: Relationships with age and body satisfaction. Body image, 10(4), 624–627. DOI: 10.1016/j.bodyim.2013.07.003 23954196

[117] Toole, A. M., & Craighead, L. W. (2016). Brief self-compassion meditation training for body image distress in young adult women. Body Image, 19, 104–112. DOI: 10.1016/j.bodyim.2016.09.001 27664531

[118] Treasure, J., Claudino, A. M., & Zucker, N. (2010). Eating disorders. The Lancet, 375(9714), 583–593. DOI: 10.1016/S0140-6736(09)61748-7 19931176

[119] Treasure, J., Duarte, T. A., & Schmidt, U. (2020). Eating disorders. The Lancet, 395(10227), 899–911. DOI: 10.1016/S0140-6736(20)30059-3 32171414

[120] Turk, F., & Waller, G. (2020). Is self-compassion relevant to the pathology and treatment of eating and body image concerns? A systematic review and meta-analysis. Clinical Psychology Review, 79. DOI: 10.1016/j.cpr.2020.101856 32438284

[121] Tylka, T. (2011). Positive psychology perspectives on body image. In T. F. Cash & L. Smolak (Eds.), Body image: A handbook of science, practice, and prevention (pp. 56–64). Guilford Press.

[122] Tylka, T. (2012). Positive Psychology Perspectives on Body Image. In T. F. Cash (Ed.), Encyclopedia of Body Image and Human Appearance (pp. 657–663). Academic Press. DOI: 10.1016/B978-0-12-384925-0.00104-8

[123] Tylka, T. (2013). Evidence for the body appreciation scale’s measurement equivalence/invariance between U.S. college women and men. Body Image, 10(3), 415–418. DOI: 10.1016/j.bodyim.2013.02.006 23523083

[124] Tylka, T. (2018). Overview of the Field of Positive Body Image. In E. Daniels, M. Gillen & C. Markey (Eds.), Body Positive (pp. 6–33). Cambridge University Press. DOI: 10.1017/9781108297653.002

[125] Tylka, T. (2019). Body appreciation. In T. Tylka & N. Piran (Eds.), Handbook of positive body image and embodiment. Oxford University Press (pp. 22–32). DOI: 10.1093/med-psych/9780190841874.003.0003

[126] Tylka, T., & Piran, N. (2019). Focusing on the positive: an introduction to the volume. In N. Piran (Ed.), Handbook of positive body image and Embodiment (pp. 1–8). Oxford University Press. DOI: 10.1093/med-psych/9780190841874.001.0001

[127] Tylka, T., & Wood-Barcalow, N. L. (2015). What is and what is not positive body image? Conceptual foundations and construct definition. Body Image, 14, 118–129. DOI: 10.1016/j.bodyim.2015.04.001 25921657

[128] van der Hoort, B., Guterstam, A., & Ehrsson, H. H. (2011). Being Barbie: The Size of One’s Own Body Determines the Perceived Size of the World. PLoS ONE, 6(5), e20195. DOI: 10.1371/journal.pone.0020195 21633503 PMC3102093

[129] Wasylkiw, L., Mackinnon, A. L., & Maclellan, A. M. (2012). Exploring the link between self-compassion and body image in university women. Body Image, 9(2), 236–245. DOI: 10.1016/j.bodyim.2012.01.007 22406200

[130] Webb, J. B., Butler-Ajibade, P., & Robinson, S. A. (2014). Considering an affect regulation framework for examining the association between body dissatisfaction and positive body image in Black older adolescent females: Does body mass index matter? Body Image, 11(4), 426–437. DOI: 10.1016/j.bodyim.2014.07.002 25079011 PMC4250321

[131] Webb, J. B., Wood-Barcalow, N. L., & Tylka, T. L. (2015). Assessing positive body image: Contemporary approaches and future directions. Body image, 14, 130–145. DOI: 10.1016/j.bodyim.2015.03.010 25910972

[132] Wertheim, E., & Paxton, S. (2011). Body image development in adolescent girls. In T. F. Cash & L. Smolak (Eds.), Body image: A handbook of science, practice, and prevention (pp. 76–84). The Guilford Press.

[133] Wood-Barcalow, N. L., Tylka, T., & Augustus-Horvath, C. L. (2010). “But I Like My Body”: Positive body image characteristics and a holistic model for young-adult women. Body Image, 7(2), 106–116. DOI: 10.1016/j.bodyim.2010.01.001 20153990

[134] Woods, H., & Proeve, M. (2014). Relationships of mindfulness, self-compassion, and meditation experience with shame-proneness. Journal of Cognitive Psychotherapy, 28(1), 20–33. DOI: 10.1891/0889-8391.28.1.20 32759128

[135] Ziser, K., Resmark, G., Giel, K. E., Becker, S., Stuber, F., Zipfe, S. L., & Junne, F. (2018). The effectiveness of contingency management in the treatment of patients with anorexia nervosa: A systematic review. European Review of Eating Disorders, 26(5), 379–393. DOI: 10.1002/erv.2590 29577487

